# The Emergence of Novel Sequence Type Strains Reveals an Evolutionary Process of Intraspecies Clone Shifting in ICU-Spreading Carbapenem-Resistant *Klebsiella pneumoniae*

**DOI:** 10.3389/fmicb.2021.691406

**Published:** 2021-08-30

**Authors:** Dongdong Zhao, Qiucheng Shi, Dandan Hu, Li Fang, Yihan Mao, Peng Lan, Xinhong Han, Ping Zhang, Huangdu Hu, Yanfei Wang, Jingjing Quan, Yunsong Yu, Yan Jiang

**Affiliations:** ^1^Department of Infectious Diseases, Sir Run Run Shaw Hospital, Zhejiang University School of Medicine, Hangzhou, China; ^2^Key Laboratory of Microbial Technology and Bioinformatics of Zhejiang Province, Hangzhou, China; ^3^Regional Medical Center for National Institute of Respiratory Diseases, Sir Run Run Shaw Hospital, Zhejiang University School of Medicine, Hangzhou, China; ^4^Department of Clinical Laboratory, National Clinical Research Center for Child Health, The Children’s Hospital, Zhejiang University School of Medicine, Hangzhou, China

**Keywords:** virulence, capsule biosynthesis locus, intraspecies shifting, recombination, carbapenem-resistant

## Abstract

Carbapenem-resistant *Klebsiella pneumoniae* (CRKP) is an urgent public health problem worldwide, and its rapid evolution in the clinical environment has been a major concern. A total of 99 CRKP isolates spreading in the intensive care unit (ICU) setting were included and subjected to whole-genome sequencing, and their sequence types (STs), serotype loci, and virulence determinants were screened based on genome data. The phylogenetic structure was reconstructed based on the core genome multilocus sequence typing method. Regions of recombination were assessed. Biofilm formation, serum resistance assays, and a *Galleria mellonella* infection model were used to evaluate strain virulence. A novel ST, designated ST4496, emerged in the ICU and spread for 6 months before its disappearance. ST4496 was closely related to ST11, with only a single-allele variant, and ST11 is the most dominant clinical clone in China. Recombination events occurred at capsule biosynthesis loci and divided the strains of ST11 and its derivative ST4496 into three clusters, including ST11-KL47, ST11-KL64, and ST4496-KL47. The phylogenetic structure indicated that ST11-KL47 was probably the origin of ST11-related strain evolution and presented more diversity in terms of both sequence similarity and phenotypes. ST4496-KL47 cluster strains presented less virulence than ST11-KL64, which was probably one of the factors preventing the former from spreading widely. In conclusion, ST4496-KL47 was probably derived from ST11-KL47 via intraspecies shifting but was less competitive than ST11-KL64, which also evolved from ST11-KL47 and developed increased virulence via capsule biosynthesis locus recombination. ST11-KL64 has the potential to be the predominant CRKP clone in China.

## Introduction

*Klebsiella pneumoniae* is a common cause of community- and hospital-acquired infections (Paczosa and Mecsas, 2016; Bengoechea and Sa Pessoa, 2019). In recent decades, carbapenem-resistant *K. pneumoniae* (CRKP) has emerged as a major clinical concern worldwide with few treatment options (Munoz-Price et al., 2013; Grundmann et al., 2017). More worrisome is the emergence of hypervirulence-associated genetic determinants in CRKP, which may lead to even higher mortality and morbidity (Gu et al., 2018).

The capsule of *K. pneumoniae* confers resistance to antimicrobial peptides, phagocytosis, and complement-mediated killing and contributes to its ability to thrive in the environment or in a host. More than 77 capsular types (K-types) have been reported in clinical isolates of *K. pneumoniae*, and recombination events involving the capsule biosynthesis (cps) region are frequently observed within several dominant sequence type (ST) clones, such as CG258 (ST258, ST512, ST11, etc.) isolates (Wyres et al., 2015; Chiarelli et al., 2020). In China, ST11 is believed to be the most dominant CRKP clone, with a prevalence greater than 60% (Zhang et al., 2017; Wang et al., 2018). The diversity of K-type capsule polysaccharides is one of the important forces driving the extensive evolution of these bacteria and has been proven to be associated with virulence (Zhao et al., 2020; Zhou et al., 2020). Enhanced virulence may lead to subclonal replacement and thus to great challenges in clinical management and infection control (Zhou et al., 2020).

In this study, we observed an intraspecies shift in CRKP at our center, and we attempted to decipher the underlying mechanisms by determining the genetic and phenotypic diversity of related groups.

## Materials and Methods

### CRKP Isolates

A common CRKP surveillance was conducted from January 1, 2017, at Sir Run Run Shaw Hospital, Zhejiang, China, which is a 1,200-bed tertiary medical center. A novel ST closely related to ST11 with a single-locus variant in *mdh* (allele: 1, C59T) was identified. Sixteen isolates with the novel ST (designated ST4496) were collected between January 2017 and May 2017. A rough *mdh* allele polymerase chain reaction analysis and subsequent sequencing screening were then applied to the isolates that were collected 2 months before January 2017 and after May 2017. Only two isolates from late December 2016 were found to harbor the novel *mdh* variant and were later identified as ST4496 by sequencing all seven alleles.

Altogether, 18 non-duplicated ST4496 CRKP isolates were detected, and the clinical data of the corresponding patients were collected from electronic medical records. All of the contemporaneous (from January to May 2017) CRKP isolates from the same intensive care unit (ICU) ward were collected and sequenced, resulting in a total of 99 CRKP isolates.

Species identification was performed by using matrix-assisted laser desorption ionization time-of-flight mass spectrometry (Bruker Daltonics, Bremen, Germany) and verified by genome sequencing. Isolates were considered carbapenem-resistant if the minimal inhibitory concentrations (MICs) of meropenem or imipenem were ≥ 4 mg/L or the MIC of ertapenem was ≥ 2, in accordance with the Clinical and Laboratory Standards Institute guidelines (2017).

The project was approved by the Ethical Review Committee of Sir Run Run Shaw Hospital (no. 20191231-20).

### Whole-Genome Sequencing

Genomic DNA was extracted using the QIAamp DNA Minikit (Qiagen, Hilden, Germany) according to the manufacturer’s recommendations and subsequently sequenced on the Illumina HiSeq X Ten platform (Illumina, San Diego, CA, United States) via a 150-bp paired-end approach. The generated short reads were *de novo* assembled using CLC Genomics Workbench 9.5.1 software, and the draft genome contigs were used for further analysis. One strain was also subjected to long-read sequencing using a MinION Sequencer (Nanopore, Oxford, United Kingdom). The *de novo* hybrid assembly of both short (Illumina) and long (Nanopore) reads was performed using Unicycler v0.4.8 in conservative mode (Wick et al., 2017).

### ST, K-Type, and Virulence Gene Analysis

Multilocus sequence typing (MLST) was performed by using the CGE database,^1^ and the novel ST was submitted to Institut Pasteur to obtain the specific ST number. The K-types were identified with Kaptive, and virulence genes were identified using the Institut Pasteur database^2^ (Wyres et al., 2016). Virulence genes [i.e., siderophore system yersiniabactin (*ybtAEPQSTUX*), salmochelin (*iroBCDN*), aerobactin (*iucABCDiutA*), and polysaccharide regulator *rmpA*/*rmpA2* genes] were chosen as genotypic biomarkers for virulence evaluation (Russo et al., 2018; Turton et al., 2019; Lan et al., 2020).

### Core Genome MLST and Recombination Analysis

All 99 assembled genomes were imported into Ridom Seqsphere + 4.1.9 (Ridom GmbH, Germany) for core genome MLST (cgMLST) analysis according to the default parameters. *K. pneumoniae* NTUH-K2044 (GenBank accession no. NC_012731.1) was used as a reference with a standard set of 2,358 genes for gene-by-gene comparisons, and the minimum spanning tree was constructed.

ST11-KL64, ST11-KL47, and ST4496 were subjected to further phylogenetic and recombination analyses. Genome alignment was established by using Snippy^3^ with the default parameters. Recombination analysis was performed by using Gubbins (Croucher et al., 2015), and the complete genome acquired by long-read sequencing was used as a reference. The region of recombination was extracted and annotated with Prokka (rapid prokaryotic genome annotation) (Seemann, 2014) and PHAST (PHAge Search Tool). Figures illustrating the phylogenetic and recombination results were produced with Phandango (Hadfield et al., 2018).

### Biofilm Formation

Microtiter dish biofilm formation assays were used to determine the capacity of biofilm formation, with minor modifications (Naparstek et al., 2014). Briefly, bacteria [10^7^ colony-forming units (CFU)/mL] were inoculated into Mueller Hinton (MH) medium in polystyrene microtiter 96-well plates (Grenier Bio-One, Frickenhausen, Germany) and incubated at 37°C for 20 h. The biofilm that formed in each well was quantified via crystal violet (Sigma, St. Louis, MO, United States) staining followed by elution with 95% ethanol and optical density (OD) measurements (OD595). Three independent cultures for each strain and quantification in four wells for each culture were performed.

### Serum Resistance Assay

Serum resistance was determined by comparing the lag phase of the growth curve in MH broth or MH broth with 10% pooled normal human serum (1 mL serum mixed with 9 mL MH broth), which was collected from healthy volunteers. Three independent cultures for each strain were grown overnight until saturation and diluted 1:1,000 in MH broth or MH broth with 10% pooled normal human serum. Three replicates of each culture were aliquoted into a flat-bottom 100-well plate (0.2 mL/well). Growth curves were determined by measuring the OD at 600 nm every 5 min for 20 h using a Bioscreen C MBR machine (Oy Growth Curves Ab Ltd., Finland). The lag phase was estimated based on the OD600 curves using an R script defined as previously described (Hua et al., 2017).

### *Galleria mellonella* Infection Model

A *Galleria mellonella* infection model was used to evaluate the virulence level among different CRKP clone groups. For strain assessment, bacteria from a freshly streaked plate were grown overnight and underwent 10-fold serial dilutions in 1 × phosphate-buffered saline (PBS). Prior to inoculation into *G. mellonella* larvae, bacterial cells were washed with PBS and then diluted to an appropriate cell density, as determined by measuring the OD at 600 nm. Groups of 30 larvae (∼200 mg; Yuejiayin, Tianjin, China) were stored in the dark at 4°C prior to use. For virulence evaluation, every larva was infected with 10-μL aliquots of 1 × 10^6^ CFU bacteria (*n* = 10) via the last left proleg by using a 10-μL Hamilton syringe. Survival was monitored every 3 h up to 24 h postinfection at 37°C. Experiments were performed in triplicate.

### Statistical Analysis

A two-tailed Mann–Whitney *U*-test was used to calculate the differences between all pairs of the ST11-KL47, ST11-KL64, and ST4496-KL47 groups regarding the median number of different core genes, biofilm formation, and the lag phase of the growth curve in MH broth or MH broth with 10% human serum. The survival rates of *G. mellonella* were evaluated using Kaplan–Meier survival curves and analyzed with the log-rank (Mantel–Cox) test. The mean numbers of bases involved in recombination were compared among these three clone groups by using one-way analysis of variance. All *p*-values ≤ 0.05 were considered statistically significant.

## Results

### Clinical Characteristics of Patients Infected With ST4496 Strains

The novel allele and ST were submitted to the administrator of the Pasteur database and assigned as ST4496 with an mdh335 allele (allelic profile of 3-3-335-1-1-1-79 for *gapA*, *infB*, *mdh*, *pgi*, *phoE*, *rpoB*, and *tonB*, respectively).

The clinical characteristics of the patients infected with ST4496 strains are presented in Table 1. All 18 isolates were collected from the ICU. Notably, none of the isolates originated from blood or primary abscesses, and the patients tended to show a good prognosis, even though five of them were discharged against medical advice because of uncontrolled underlying conditions. Moreover, the relatively good outcomes of the majority of the patients receiving no effective antimicrobials for CRKP indicated a colonized status of the strains rather than an infectious status. The non-invasive clinical manifestations indicated a hypovirulent character of the ST4496 CRKP.

**TABLE 1:** Clinical characteristics of the patients infected with ST4496 CRKP.

Isolates	Time of isolation	Origin	Department	Underlying conditions	Clinical outcome	Antimicrobials
NST-1	21/12/2016	Bile	ICU	Severe acute pancreatitis	Improved	Cefoperazone and sulbactam, tigecycline
NST-2	26/12/2016	Ascites	ICU	Pseudomyxoma peritonei	Improved	Imipenem
NST-3	05/01/2017	Sputum	ICU	Colon cancer	Improved	Amoxicillin and clavulanic acid
NST-4	09/01/2017	Urine	ICU	Bladder cancer	Improved	Cefoperazone and sulbactam
NST-5	16/01/2017	Sputum	ICU	Sepsis	Improved	Cefoperazone and sulbactam, tigecycline
NST-6	26/01/2017	Ascites	ICU	Hepatic carcinoma	Improved	Imipenem
NST-7	26/01/2017	Sputum	ICU	Heart failure	Improved	Moxifloxacin, imipenem
NST-8	02/02/2017	Urine	ICU	Deep vein thrombosis, GI bleeding	Improved	Cefoperazone and sulbactam
NST-9	02/02/2017	Purulent discharge from surgical site	ICU	Femoral artery embolectomy, atrial fibrillation, heart failure	Against-advice discharge	Amoxicillin and clavulanic acid, cefepime
NST-10	02/02/2017	Sputum	ICU	Brain ischemia	Improved	Piperacillin and tazobactam
NST-11	11/03/2017	Sputum	ICU	Esophageal cancer, pyothorax, respiratory failure	Against-advice discharge	Tigecycline, meropenem
NST-12	22/03/2017	Sputum	ICU	Brain hemorrhage	Improved	Meropenem
NST-13	22/03/2017	Sputum	ICU	GI bleeding	Improved	Piperacillin and tazobactam
NST-14	23/03/2017	Sputum	ICU	Cerebral infarction, pulmonary infection	Against-advice discharge	Amoxicillin and clavulanic acid, cefepime
NST-15	02/04/2017	Sputum	ICU	Brain hemorrhage	Improved	Cefoperazone and sulbactam
NST-16	05/04/2017	Ascites	ICU	Acute generalized suppurative peritonitis, exploratory laparotomy	Improved	Imipenem, Tigecycline
NST-17	10/04/2017	Purulent discharge from surgical site	ICU	Esophageal perforation from foreign body, mediastinal abscess	Against-advice discharge	Imipenem
NST-18	02/05/2017	Urine	ICU	Malignant tumor of the meninges, lymphoma suspected	Against-advice discharge	Cefoperazone and sulbactam

### cgMLST for Contemporaneous CRKP

Altogether, 99 contemporaneous isolates were included in the cgMLST analysis and the subsequent phylogenetic analysis. K-type was also considered as capsule synthesis loci are recombination hotspots in multidrug-resistant *K. pneumoniae* and are associated with virulence.

**FIGURE 1:**
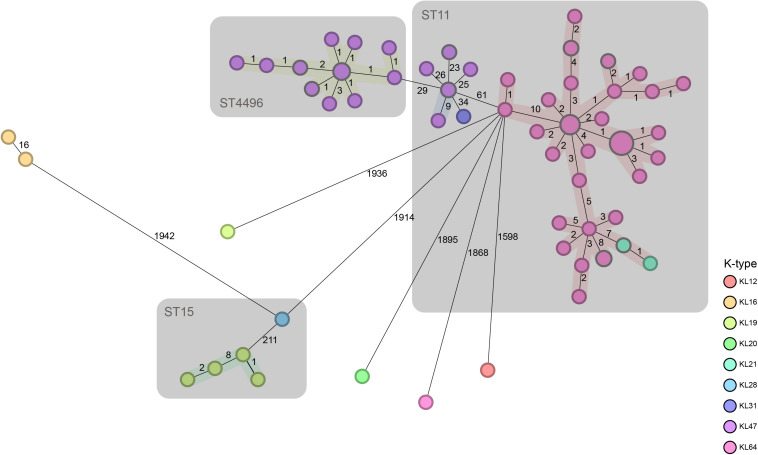
Minimum spanning tree based on the cgMLST profiles of contemporaneous CRKP strains. cgMLST profiles are represented by circles, and the size of the circle is proportional to the number of isolates with an identical cgMLST profile. The color of each circle is encoded by the K-type, and the length of lines connecting the cgMLST profiles is proportional to the number of core gene differences between circles. A gray zone surrounds a group of circles that share the same sequence type.

The minimum spanning tree based on the cgMLST profiles is presented in Figure 1. The results showed that ST11 (70/99) accounted for the majority of CRKP isolates, among which KL64 and KL47 were the overwhelmingly dominant serotypes. ST4496 (18/99) was the second most common ST, and all of the isolates with this ST belonged to the KL47 serotype. There were five ST15 isolates, and the remaining six isolates included five distinct STs. ST4496 and ST11 were closely related to each other but displayed relatively long distances from other STs. ST4496 showed a shorter phylogenetic distance from ST11-KL47 strains than from ST11-KL64 based on the core gene difference. As ST4496 strains have never previously been detected at this center or reported in any other studies, it is logical to believe that they may have originated from ST11-KL47.

**FIGURE 2:**
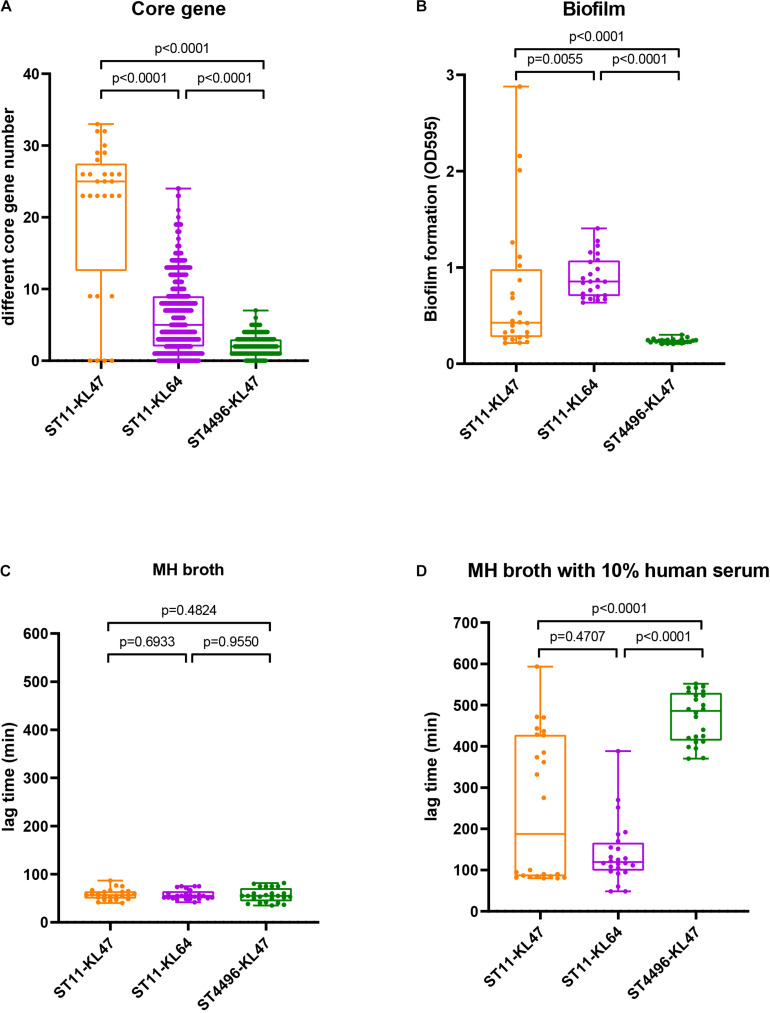
Genetic evidence and phenotypic manifestations among the ST11-KL47, ST11-KL64, and ST4496-KL47 groups: **(A)** The distribution of different core gene numbers between each pair of strains within the group; **(B)** biofilm formation (OD595); **(C)** lag phase in MH broth; **(D)** lag phase in MH broth with 10% human serum. The middle lines represent the median value for each group. Error bars indicate the region from the lowest to highest values in each group.

ST4496-KL47 (*n* = 18), ST11-KL64 (*n* = 58), and ST11-KL47 (*n* = 8) were selected for further analysis, as these three groups were very closely related to each other according to cgMLST and were the predominant CRKP groups. Notably, ST11-KL47 exhibited greater genetic diversity than the other two clusters; among the clusters, the median number of different core genes between each pair of strains within the group was 25 in ST11-KL47 vs. 5 in ST11-KL64 and 2 in ST4496-KL47, and these differences were significant (both *p* < 0.0001; Figure 2A).

### Phylogenetic and Recombination Analysis

**FIGURE 3:**
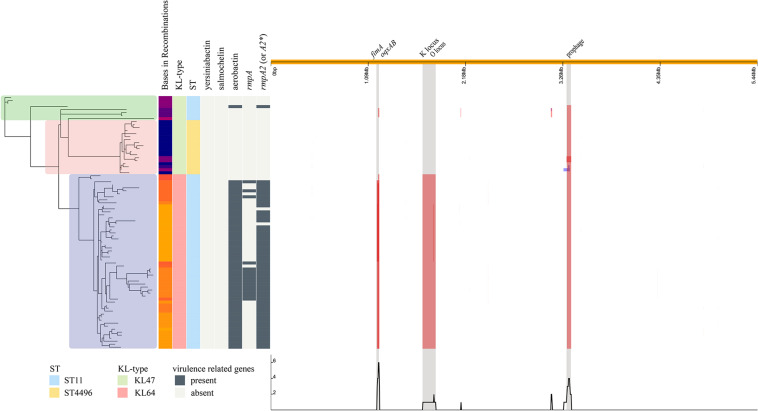
Phylogenetic structure combined with the molecular characteristics and the regions of recombination in the ST11-KL47, ST4496-KL47, and ST11-KL64 strains. The SNP-based phylogenetic tree is shown to the left; green zone, ST11-KL47; pink zone, ST4496-KL47; blue zone, ST11-KL64. The distributions of bases in recombinations, KL type, ST type, and some virulence-related genes are shown in the middle. The three major regions of recombination are shown to the right, and the largest region corresponds to the K locus and O locus.

Further phylogenetic analysis was performed on 84 isolates and revealed three distinct clusters, corresponding to ST11-KL47, ST4496-KL47, and ST11-KL64 (Figure 3). The strains in cluster ST11-KL47 had longer branches, continuing to show more genetic diversity than the other two clusters. The mean number of bases involved in recombination in ST11-KL64 was 270,666.7, whereas 56,866.6 and 10,879.2 bases were found in ST11-KL47 and ST4496-KL47, respectively (*p* < 0.0001). The three major regions of recombination were annotated, and the largest region corresponded to the K locus and O locus (149,594 bases), deciphering the K-type and LPS shifts. The other two regions were *fimA*/*oqxAB* (27,879 bases) and the prophage sequence (45,116 bases).

### Virulence Genes

Several key virulence genes were screened, including the capsular polysaccharide regulator *rmpA* or *rmpA2*, and siderophore systems (yersiniabactin, salmochelin, and aerobactin). Yersiniabactin was the most common of these genes and was found in all strains, whereas salmochelin was not found in three lineages, ST11-KL47, ST11-KL64, and ST4496-KL47. However, great differences in other virulence genes were observed among the three lineages. Among ST11-KL64 isolates, not only did the majority (56/58, 96.6%) possess aerobactin*/rmpA2^∗^* genes [*rmpA2*^∗^ indicates a frameshift mutation compared to empirical *rmpA2* gene (Zhou et al., 2020)], but a relatively high proportion (15/58, 25.9%) also carried the *rmpA* gene. Only one isolate of ST11-KL47 contained aerobactin and the empirical *rmpA2* gene. The ST4496 isolates carried no targeted virulence genes beyond the yersiniabactin gene cluster *ybtAEPQSTUX*.

### Phenotypic Assessment

**FIGURE 4:**
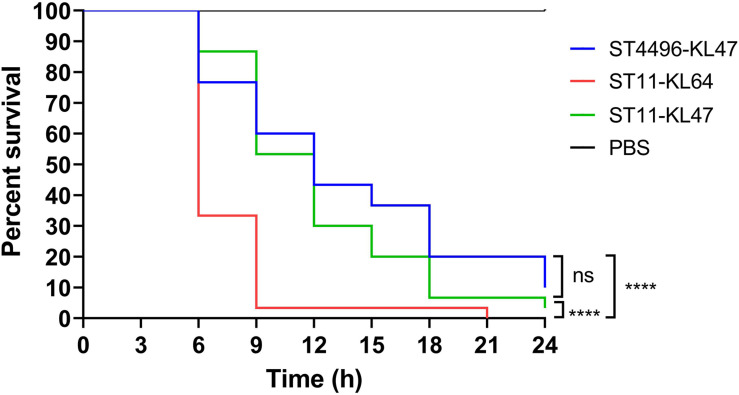
Percent survival curve of *G. mellonella* infected with ST11-KL64 isolates (red line), ST11-KL47 isolates (green line), and ST4496-KL47 (blue line). ns, no significant difference; ****p < 0.0001.

All eight ST11-KL47 isolates were included in the following phenotypic analysis. For biofilm formation and serum resistance assays, eight isolates were randomly chosen as representatives for the phenotypic assessment of ST4496-KL47 and ST11-KL64, respectively. In the biofilm formation assay, the OD measurements indicated that ST4496-KL47 isolates produced the least biofilm, whereas ST11-KL64 isolates produced the most biofilm. The differences between the three groups were significant (median OD595 values in ST11-KL47, ST11-KL64, and ST4496-KL47 of 0.4264, 0.8531, and 0.2340, respectively, Figure 2B). Greater diversity in biofilm formation was observed for ST11-KL47 than for ST11-KL64 and ST4496-KL47. In the serum resistance assay, similar lag phases were detected for the three groups in the MH broth (Figure 2C). However, a significantly prolonged lag phase was observed for ST4496-KL47 and some of the ST11-KL47 isolates in MH broth with 10% human serum relative to ST11-KL64. The differences between ST11-KL47 and ST4496-KL47 or between ST11-KL64 and ST4496-KL47 were significant (*p* < 0.0001). Similarly, greater variation in the lag phase in MH broth with 10% pooled normal human serum was observed in ST11-KL47 (Figure 2D). Furthermore, we generated a *G. mellonella* infection model to evaluate the virulence among these three groups. The survival percentage of *G. mellonella* larvae in the ST11-KL64 group was significantly lower than that in the ST11-KL47 or ST4496-KL47 group, indicating that the ST11-KL64 isolates had the highest virulence compared to other KL47 serotype isolates (Figure 4).

## Discussion

In this study, a novel ST with one single-locus variant in *mdh* emerged in the ST11 CRKP and was assigned ST4496. Unlike other successful descendants (e.g., ST258) of ST11, ST4496 did not show successful persistence or dissemination in the hospital setting. The characteristics of patients infected with ST4496 indicated a hypovirulent nature, as ST4496 strains mostly presented colonization rather than infection, and no bloodstream infection involving ST4496 was detected. We presumed that hypovirulence was mainly responsible for the restriction of the spread of ST4496 after it caused occasional outbreaks in the hospital setting for a couple of months.

There are growing reports of convergent evolution of resistance and virulence in *K. pneumoniae*. Although convergence can result from hypervirulent strains gaining multidrug resistance (MDR) elements or MDR strains gaining virulence elements, MDR clones are more likely to acquire virulence genes than hypervirulent clones are to acquire resistance genes (Wyres et al., 2019). Following sporadic reports of isolates showing the phenomenon of convergence in clinical isolates, there has recently been growing evidence of increasing virulence in the CRKP population, especially in the dominant ST11 clone in China (Shen et al., 2020; Zhao et al., 2020; Zhou et al., 2020). To reveal the genotypic and phenotypic basis of this convergence, novel ST4496 isolates and contemporary CRKP isolates were included in the present study. The K-type was also investigated because it has been proven to be associated with virulence, and recent evidence showed that the previously prevalent ST11-KL47 has been gradually replaced by ST11-KL64, which was derived from an ST11-KL47–like ancestor through recombination (Zhao et al., 2020; Zhou et al., 2020).

From the cgMLST analysis and the derived minimum spanning tree, we can see that ST4496-KL47, ST11-KL64, and ST11-KL47 were closely related to each other and were the predominant CRKP groups. The relationships of the three groups were further confirmed by single-nucleotide polymorphism (SNP)–based phylogenetic and recombination analysis. ST11-KL47 is much more diverse genetically. Since it has been reported that enhanced virulence promotes the replacement of ST11-KL47 by ST11-KL64 (Zhou et al., 2020), it is logical to presume that different levels of virulence may be one of the factors leading to the different fates of the three CRKP groups, that is, the disappearance of ST4496-KL47, the decrease in ST11-KL47, and the proliferation of ST11-KL64, although both ST4496-KL47 and ST11-KL64 may have evolved from ST11-KL47.

This assumption was supported by the fact that enhanced virulence of ST11-KL64 and weakened virulence of ST4496-KL47 were observed both genetically and phenotypically. Genetically, ST11-KL64 has the most virulence genes, and ST4496-KL47 has the fewest virulence genes. As previously reported, *iuc* (the aerobactin locus) is important for the production of siderophores to promote iron acquisition, and some researchers have even considered aerobactin as an indicator of hypervirulent *K. pneumoniae* (Zhang et al., 2016). The presence of *rmpA/A2*, which encodes regulators of mucoid phenotype genes, is strongly associated with the phenotype of hypermucoviscosity. Positivity for *iuc*, *rmpA*, and *rmpA2* (or *rmpA2*^∗^) may indicate the presence of a virulence plasmid (Wyres et al., 2020).

Virulence levels were further determined by phenotypic tests. First, the observation of biofilm formation supported bacterial adhesion to indwell medical devices and potentially better survival in adverse environmental conditions, which may lead to drug ineffectiveness and failure to eradicate infection (O’Toole et al., 2000; Zheng et al., 2018). Previous studies have shown that biofilm formation ability is associated with genetic factors such as *iucA* and *rmpA/rmpA2* in *K. pneumoniae* (Zheng et al., 2018). Second, a significantly prolonged lag phase was observed for ST4496-KL47 and some of the ST11-KL47 isolates in MH broth with 10% human serum. In fact, some ST4496-KL47 and ST11-KL47 isolates failed to grow in MH broth with a higher concentration of human serum (20%) over a 20-h period (data not shown). A shorter lag phase may indicate a relatively high serum resistance ability of ST11-KL64. Finally, the highest virulence level in ST11-KL64 isolates was also observed based on the *G. mellonella* infection model, implying that this clone was more likely to cause pandemic outbreaks than other KL47 serotype isolates.

Relative to ST4496-KL47 and ST11-KL64, diversity is much higher in ST11-KL47 based on both genetic evidence and phenotypic findings. This is consistent with the fact that this group is much more ancient and has therefore had a higher chance to evolve and develop greater diversity.

Our findings are relevant for understanding the risk of carbapenem-resistant hypervirulent *K. pneumoniae* strains as highly virulent MDR strains may be able to persist and be disseminated in hospital settings and may even spread to community settings, leading to public health disasters (Chen and Kreiswirth, 2018). The early detection and containment of spreading through comprehensive infection control measures could be the most feasible solution to this problem.

This work provides a glimpse of intraspecies shifts from one hospital, and a preliminary investigation has been conducted. Certainly, more studies are needed to verify this assumption. To better understand the mechanisms of evolution, we should pay attention to “unsuccessful” evolution as well as successful evolution. Thus, unsuccessful ST4496 could be an excellent reference when studying the evolution of *K. pneumoniae*.

## Conclusion

The present study provided further evidence that virulence enhancement in ST11-KL64 was the reason for intraspecies replacement, as further demonstrated by the disappearance of ST4496-KL47, with weakened virulence. The results indicate that ST11-KL64 has the potential to be the predominant CRKP strain in China, and it seems that the convergent evolution of virulence and resistance in *K. pneumoniae* is inevitable under current antimicrobial strategies and infection control policies.

## Data Availability Statement

The datasets presented in this study can be found in online repositories. The names of the repository/repositories and accession number(s) can be found below: NCBI BioProject, accession numbers: PRJNA747841 and PRJEB34922.

## Author Contributions

DZ and QS participated in the design of the study, performed the bioinformatics analysis, and drafted the manuscript. DH, LF, and YM performed Genomic DNA extraction and prepared for illumine sequencing. PL and JQ participated in the bioinformatics analysis and generated some figures. XH and PZ performed biofilm formation assay. HH and YW performed serum resistance assay. YJ and YY conceived of the study, participated in its design and coordination and helped to draft the manuscript. All authors read and approved the final manuscript.

## Conflict of Interest

The authors declare that the research was conducted in the absence of any commercial or financial relationships that could be construed as a potential conflict of interest.

## Publisher’s Note

All claims expressed in this article are solely those of the authors and do not necessarily represent those of their affiliated organizations, or those of the publisher, the editors and the reviewers. Any product that may be evaluated in this article, or claim that may be made by its manufacturer, is not guaranteed or endorsed by the publisher.

## Footnotes

1
http://www.genomicepidemiology.org/

2
https://bigsdb.pasteur.fr/klebsiella/klebsiella.html

3
https://github.com/tseemann/snippy
